# Involvement of SARA in Axon and Dendrite Growth

**DOI:** 10.1371/journal.pone.0138792

**Published:** 2015-09-25

**Authors:** Cristina Isabel Arias, Sebastián Omar Siri, Cecilia Conde

**Affiliations:** 1 Laboratorio Neurobiología, INIMEC-CONICET, Córdoba, Argentina; 2 Universidad Nacional de Córdoba, Córdoba, Argentina; 3 Instituto Universitario de Ciencias Biomédicas de Córdoba, Córdoba, Argentina; NHLBI, NIH, UNITED STATES

## Abstract

SARA (Smad Anchor for Receptor Activation) plays a crucial role in Rab5-mediated endocytosis in cell lines localizing to early endosomes where it regulates morphology and function. Here, we analyzed the role of SARA during neuronal development and tested whether it functions as a regulator of endocytic trafficking of selected axonal and membrane proteins. Suppression of SARA perturbs the appearance of juxtanuclear endocytic recycling compartments and the neurons show long axons with large growth cones. Furthermore, surface distribution of the cell adhesion molecule L1 in axons and the fusion of vesicles containing transferring receptor (TfR) in dendrites were increased in neurons where SARA was silenced. Conversely, SARA overexpression generated large early endosomes and reduced neurite outgrowth. Taken together, our findings suggest a significant contribution of SARA to key aspects of neuronal development, including neurite formation.

## Introduction

In mammal cells, endocytic membrane traffic plays an essential role in delivering membrane components, receptor-associated ligands and solute molecules to intracellular destinations. This requires significant coordination between spatially segregated sorting compartments that function to determine the cellular fate of cargos. After internalization, a cargo is transported to early endosomes (EE) where sorting decisions are made [1]: proteins targeted for degradation shift to late endosomes and lysosomes, whereas proteins recycled to the cell surface through recycling endosomes (RE) are subject to slow recycling, or fast recycling if travelling directly from early endosomes for later reinsertion into the plasma membrane [2–3]. The fate of the endocytic cargo is determined by the activity and molecular nature of the endosomal sorting machinery.

The endosomal pathway is known to play a decisive role in many neurodevelopment processes, including migration, polarization and synaptic function [4–7]. Neurons are among the best examples of polarized cells, having two functionally different structural domains: a single long axon, and multiple short highly branched dendrites. In neurons, the regulation of endosomal trafficking is particularly complex, since the generation of asymmetric domains requires specialized membrane trafficking not only to promote neurite outgrowth but also to ensure differential distribution of components to the axonal or somatodendritic domains [8–11]. Dysfunction of proteins involved in endocytic trafficking has been linked to the development of neurodegenerative diseases, implicating at the membrane trafficking control machinery as a critical factor in neuron function [12–16].

SARA is a FYVE protein (Fab1, YOTB, Vac1 and EEA1, [17]) that binds to PI3P (phosphatidylinositol 3-phosphate), is highly enriched in endocytic membranes and is involved in membrane trafficking. [18]. SARA also contains a Smad-binding domain (SBD) required for the interaction with the transcription factors Smad2 and Smad3 [19] and a C-terminal region that interacts with the type I TGFβ receptor (TGFβ-RI) [17]. It has been suggested that SARA has a crucial function in the recruitment of Smad to the TGFβ receptor, ensuring appropriate subcellular localization of the activated receptor-bound complex. The FYVE domain directs the ligand TGFβ to EE, where it interacts with both TGFβ receptors and Smads [17]. Recent data suggests that SARA is dispensable for functional TGFβ-mediated signaling, because in various B-cell lymphomas no correlation was found between SARA expression and the levels of TGFβ-induced phosphorylation of Smads. Moreover, knockdown of SARA in HeLa cells does not interfere with TGFβ-induced Smad activation, Smad nuclear translocation, or induction of TGFβ target genes [20]. These data suggest that SARA may regulate other events. For example, it has been shown that SARA overexpression causes enlargement of EE, and significantly delays transferrin recycling. These alterations resemble the defects caused by overexpression of the Rab5 mutant (Rab5Q79L) and suggest that SARA plays an important functional role downstream of Rab5-regulated endosomal trafficking [21, 22]. Interestingly, SARA also interacts with ubiquitin ligase RNF11, participates structurally and functionally in the ESCRT (endosomal sorting complexes requi**r**ed for transport) and regulates degradative EGFR trafficking [23].

Recently, Chang et al. have suggested a protective role of SARA in skin carcinogenesis, showing that SARA is not involved either in the activation process of TGF-β signal transduction or mouse development [24]. Moreover, SARA has been proposed as a novel vesicle-tethering molecule capable of interacting with membrane proteins such as rhodopsin and syntaxin 3 in axonemal vesicles [25], suggesting that SARA may play a role in neuronal morphogenesis. In the present study, we analyzed the consequences of the expression or knockdown regulation of SARA on neuronal development. We provide novel evidence suggesting a key role for SARA in several neuronal morphogenetic events, including neurite formation, development and polarized growth. We propose that some of these functions may be linked to the role of SARA in regulating the trafficking of membrane components between EE and RE, both in axons and in dendrites.

## Materials and Methods

### DNA constructs

Expression vectors encoding SARAsh-HcRed, SARAsh-GFP and scrambled control-sh plasmid were described previously [25]. The SARA-GFP or SARA-Flag plasmids were described in Hu et al. [21]. Rab8-GFP, Rab11a-GFP and Rab5-GFP were generously gifted by Dr J.L. Daniotti (National University of Córdoba, CIQUIBIC); these were generated by Dr J.L. Daniotti, Dr M. Colombo (National University of Cuyo) and Dr. J. Bonifacino (NICHD, National Institutes of Health, Bethesda, MD, USA), respectively. Rab4-GFP was a kind gift from Dr. G. Yudowski (Departments of Psychiatry, University of California at San Francisco) and TfR-GFP from Dr. Thierry Galli (INSERM, Institut Jacob Monod, France).

### Antibodies

The following primary antibodies were used in this study: mAb against Tau protein, an axonal marker (clone Tau-1) and mAb against MAP2, a dendritic marker (clone AP-20) (both were a generous gift of Dr. L. I. Binder, Northwestern University, Chicago, USA) diluted 1:1000 and 1:400, respectively; mAb against tyrosinated α-tubulin (Tyr-Tubulin; Sigma Ch. Co. USA) diluted 1:8000 for IF or 1:10000 for WB; polyclonal antibody (pAb) against SARA (H-300) (sc-9135, Santa Cruz Biotechnology, Inc. CA, USA) diluted 1:20 for IF or 1:200 for WB; an affinity purified rabbit polyclonal antibody raised against L1 (provided by Dr. Hiroyuki Kamiguchi); mAb against GFP (ROCHE) diluted 1:400 for IF and 1:1000 for WB; rabbit polyclonal antibody raised against Flag (Sigma Aldrich) diluted 1:500 for IF or 1:2000 for WB and a rabbit polyclonal antibody against LIMK1 (Santa Cruz Biotechnology) diluted 1:1000 for WB. For some experiments rhodamine-phalloidin (1:1000; Molecular Probes, Eugene, OR, USA) was used to stain filamentous actin (F-actin).

### Culture, transfection, immunofluorescence and imaging

Pregnant rats (Wistar, Charles River 251 Ballardvale Street, Wilmington, MA01887) were obtained from the institutional (INIMEC-CONICET) specific pathogen free (SPF) vivarium. All animal procedures and care were approved by the institutional animal care committee (INIMEC-National Research Council and Universidad Nacional de Córdoba, Argentina) and the National Department of Animal Care and Health (SENASA-Argentina). Efforts were made to minimize animal suffering and to reduce the number of animals used.

Embryonic day-18 rat embryos (euthanized by CO_2_ gas in chamber with gas inflow precisely regulated) were used to prepare primary hippocampal cultures as described [26–28]. SARA-KO mice were generated as described by Chang et al, 2014 [24] and were generously provided by Dr C.H. Sung (Cornell University, USA). Transient transfection of cultured neurons was performed with Lipofectamine 2000 [26] and the constructs used at concentrations ranging from 2–4 μg/ml. Neurons were fixed 16 h later (for overexpression experiments) or 20–24 h later (for silencing experiments) with 4% PFA in 4% sucrose-containing PBS, and permeabilized in 0.2% Triton X-100 in PBS for 5 min prior to antibody incubation. CHO-K1 cells were cultured in high glucose DMEM containing L-glutamine, supplemented with 10% fetal bovine serum, penicillin and streptomycin (Invitrogen, Carlsbad, CA).

Cells were visualized using a conventional (Zeiss Pascal) inverted confocal microscope. Images were processed using Adobe Photoshop. Neuronal shape parameters were evaluated as described by Bisbal et al. [29]. Briefly, montages showing the complete neuronal arbor of transfected neurons were created from confocal images (maximal projections) acquired through a 60 X 1.4 NA oil objective lens and the number and length of neuritic processes measured using ImageJ software. Manders coefficients, m1 and m2, are proportional to the amount of fluorescence of the colocalizing objects in each component of the image, relative to the total fluorescence in that component. From the distribution of green (EEA1) / red (SARA) or red (SARA) / green (EEA1) pixels, m1 and m2, respectively, were calculated. Statistical analysis was performed with Statistica software (StatSoft, v8.0). Data are presented as the mean ± s.e.m. from at least three representative independent experiments for each DNA construct. Differences among experimental groups were analyzed by one-way ANOVA and post-hoc Tukey test. Statistical significance was defined as p< 0.05, 0.01 or 0.001.

### Western blot assays

Levels of endogenous SARA, ectopically expressed SARA and tubulin after shRNA treatment in Chinese hamster ovary (CHO-K1) cells or in cultured hippocampal pyramidal neurons were analyzed by Western blotting as described [27, 30]. Immunoblotting assays using extracts of transfected CHO-K1 cells or brain lysates were carried out essentially as described by Bisbal et al [30].

### Analysis of cell-surface proteins

To label cell-surface proteins, CHO-K1 cells were washed three times with ice-cold PBS, pH 8, and then incubated with the membrane impermeable sulfo-NHS-biotin (Pierce, Rockford, IL, USA) at a final concentration of 0.3 mg/ml in PBS, pH 8, at 4°C for 20 min. After biotinylation, the cultures were washed with ice-cold PBS and harvested with RIPA buffer. Biotinylated proteins were collected with avidin-agarose beads (Pierce) and analyzed by Western blot with indicated antibodies.

### Time-lapse video microscopy, total internal reflection fluorescence microscopy (TIRFM) and Fluorescence recovery after photobleaching (FRAP)

For time-lapse fluorescence microscopy and total internal reflection fluorescence microscopy (TIRFM), transfected cells were cultured in special Petri dishes [31]. After transfection, the dishes containing the attached cells were placed on a Harvard microincubator located on top of the stage of a fully motorized Nikon TE-2000 E inverted microscope equipped for differential interference contrast, epifluorescence, and TIRFM. For conventional time-lapse fluorescence microscopy, cells were imaged using a 60X or a 100X oil-immersion objective, an ORCA II ER camera (Hamamatsu) and MetaMorph software (Molecular Devices) [32]. We used TIRFM to image single vesicular insertion events under low intensity and superficial light exposure conditions that minimize phototoxicity [33]. Cells were visualized with a 60X 1.45 NA objective, equipped for through-the-objective TIRF illumination using a 488 nm argon laser. Neurons were imaged in Neurobasal medium supplemented with 30 mM HEPES buffer, pH 7.2, and maintained at 37°C. Time-lapse sequences were acquired at a continuous rate of 2 or 5 frames/s during 2–4 min. Pyramidal neurons were selected by morphological criteria (in wide-field images) before imaging in TIRFM mode. Live images shown represent raw data with simple background subtraction of the averaged blank field intensity. Fusion events were detected semiautomatically using maximum pixel intensity and centroid analysis to define sites of insertion [33]. FRAP experiments were performed in CHO-K1 cells previously transfected with Rab11-GFP and HcRed or sh-SARA and analyzed 20 h later. These experiments were performed in an Olympus FluoView FV1000 confocal microscope (Olympus, Japan) using a 20X UPlanApo oil immersion/0.8 NA objective. In these experiments, the pinhole was fully opened in order to minimize changes in fluorescence detection due to Z-drift occurring during time lapse imaging. Before performing FRAP experiments, we standardized bleaching protocols to avoid significant reversible photobleaching using standard protocols [34]. FRAP methodology and quantitative image analyses were those previously described [35–36].

## Results

### SARA distributes in somatodendritic and axonal compartments in hippocampal neurons

Most of the experiments designed to study the function of SARA have been performed in cultured cells (HEK and MDCK cell lines) [21] or human mesangial cells [37]. However, little is known about the subcellular distribution of SARA in neurons. We sought to address this issue using neuronal cultures prepared from rat embryonic hippocampi, a widely used model for studying neuronal morphogenesis [38].

**Fig 1:**
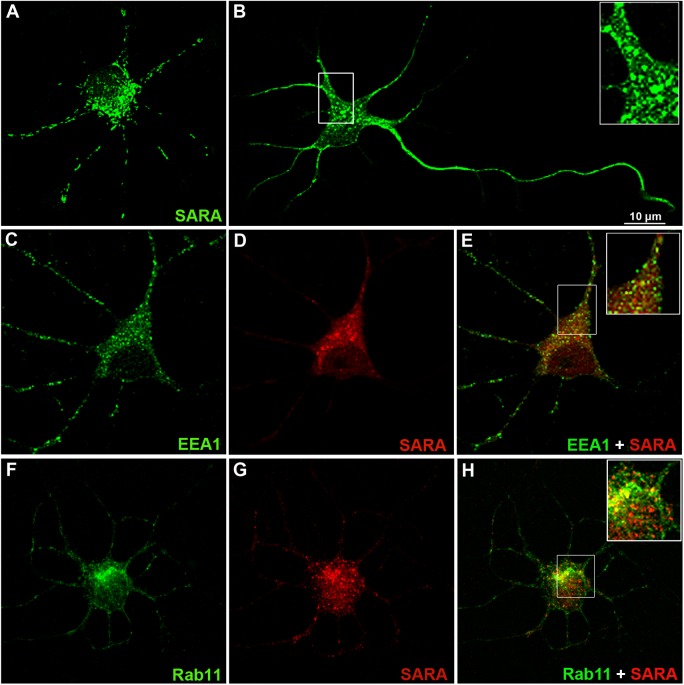
SARA subcellular distribution in young cultured hippocampal pyramidal neurons. (A) Confocal image showing an example of stage 2 neurons (1DIV) or (B) stage 3 neurons (2-3DIV) immunostained for SARA (green). Note that SARA immunolabeling is present in the soma, in all types of neurites (minor process and axon) and that it extends into neuritic tips. SARA is associated with early and recycling endosomal compartments. (C-H) Neurons double-labeled, using antibodies against EEA1 (green, C) and SARA (red, D) or Rab11 (green, F) and SARA (red, G). The SARA-labeled punctae displayed partial colocalization with the early endosomal marker (E, inset) or recycling endosomal marker (H, inset). Different heterogeneous populations of endosomes were observed: one containing only EEA1 or Rab11, a second with EEA1 or RAb11 and SARA, and a third containing only SARA endosomes. All cells were imaged by confocal microscopy. Data is typical of over 10–20 neurons imaged for each condition.

First, we examined the cellular localization of SARA in young cultured hippocampal neurons (during the first 48 hours in vitro), when they either display a symmetric array of short neurites (minor undifferentiated processes, stage 2, Fig 1A) or have already developed an axon-like neurite (stage 3, Fig 1B). Staining with a SARA antibody revealed punctated (tubulo-vesicular), endosome-like labeling throughout the neuron. Confocal fluorescence microscopy revealed that, in neurons in stages 2 and 3, SARA is evenly distributed throughout the cell body, minor neurites, the axon and growth cones (Fig 1A and 1B). The SARA-labelled punctae displayed a partial but significant colocalization with the early endosomal markers EEA1 (Fig 1C–1E) or Rab5 (not shown) and with the recycling endosome marker, Rab11 (Fig 1F–1H). Quantitative estimation of colocalization was further performed using the JACoP plugin of ImageJ. The values for Pearson correlation (r) revealed a positive correlation between SARA and EEA1or Rab11 (0.70 and 0.67, respectively). The Manders coefficients, m1and m2 [39], had values of 0.80 and 0.65, respectively, indicating that at least 65% of SARA-positive endosomes colocalize with EEA1-positive endosomes. However, m1 and m2 values for Rab11 were 0.77 and 0.8, indicating that nearly 80% of SARA endosomes colocalize with Rab11-positive endosomes.

To avoid the use of the high concentrations of antibodies required to detect endogenous Rabs, as a second approach, neurons were transiently transfected with GFP tagged Rabs. These GFP fusioned for Rab4, Rab5, and Rab11 correctly localized to their target membranes [40]. Confocal images of neurons expressing Rab5-GFP and stained for endogenous SARA revealed that both proteins partially colocalized (S1 Fig). The quantitative analysis showed results for SARA and EEA1 similar to those we previously found using antibodies to detect endogenous Rabs (r = 0.76; m1 and m2 = 0.77).

Rab4 is a protein associated with sorting endosomes, a tubular mature compartment of EE [41–42]. Our results show that Rab4-GFP partially colocalizes with endogenous SARA in vesicle-like structures (S1 Fig). The colocalization indexes obtained were r = 0.8, m1 = 0.86 and m2 = 0.87 for Rab4-GFP and endogenous SARA.

Our results above described revealed that, whereas some neuronal domains have endosomes with partial or good colocalization indexes between EEA1 or Rab5 and SARA, other domains show endosomes differentially enriched with either EE markers or SARA. This suggests that the remaining endosomes non-colocalizing would be either in the same endosome but in a different domain [23, 40] or in different endosomes, and they would probably have different functions [43].

In summary, these experiments show that SARA is distributed in neurons in all the types of neurites where it partially localizes to different functional domains of EE and RE. These observations also raise the possibility of SARA having a role in neuronal development.

### SARA suppression changes recycling endosome distribution

**Fig 2:**
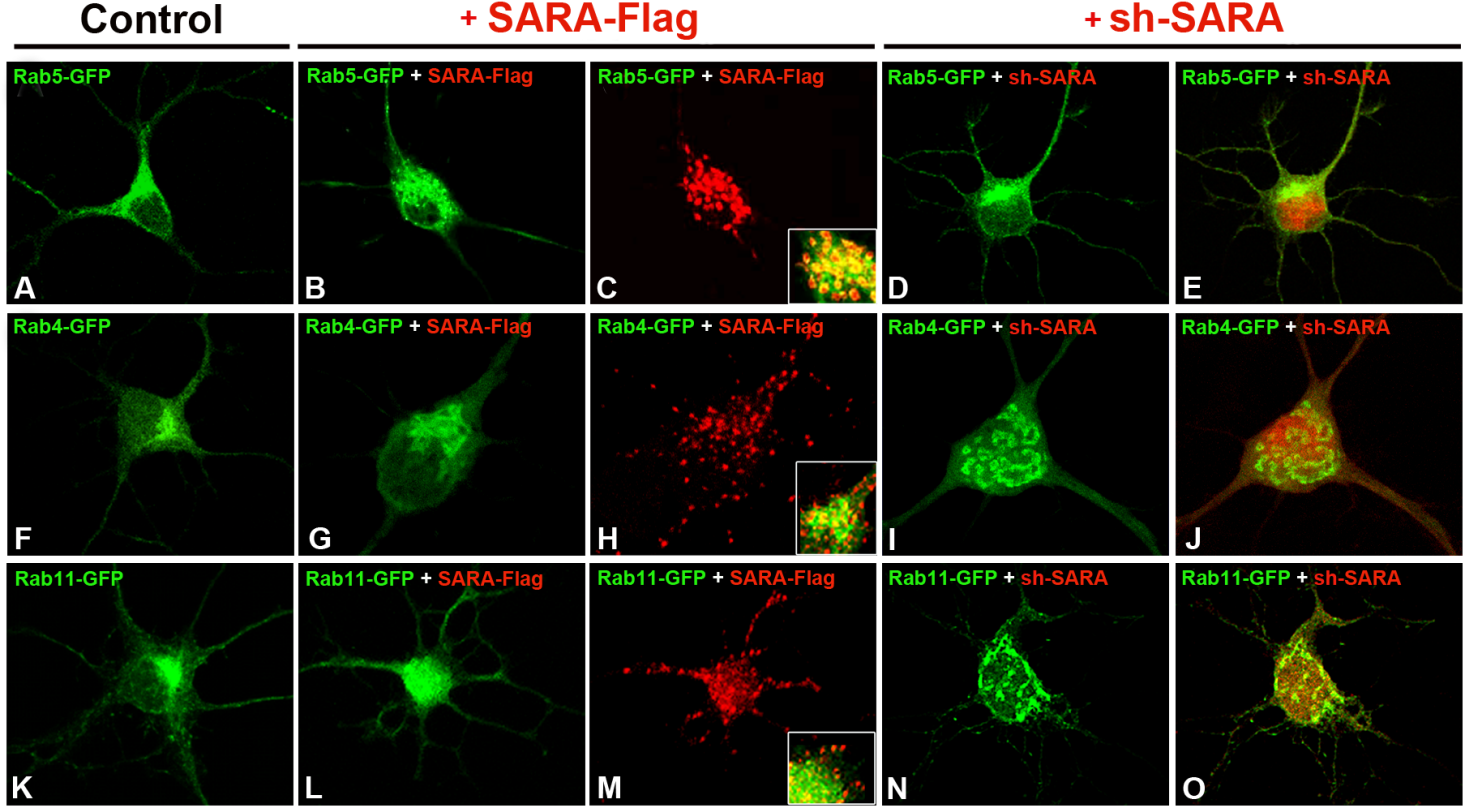
Characterization of Rabs distribution in neurons with modified SARA expression. Hippocampal pyramidal neurons at 7DIV were transfected with (A) Rab5-GFP, (F) Rab4-GFP or (K) Rab11-GFP where usual Rabs localization was observed. In neurons cotransfected with Rab5-GFP + SARA-Flag (B-C, inset), Rab5 labeling was extensively colocalized with SARA-Flag on enlarged early endosomal membranes; (G-H) no obvious alterations were observed for Rab4 endosomes after SARA overexpression. (L-M) Images showing intracellular distribution of Rab11-positive endosomes when SARA was overexpressed. Images of neurons transfected with (I-J) Rab4-GFP or (N-O) Rab11-GFP and cotransfected with sh-SARA (red); Rab4- and Rab11-positive recycling endosomes were distributed occupying the neuronal perikaryon area compartment compared to their distribution in controls (I vs F; N vs K). (D-E) No changes were observed in Rab5-GFP neurons cotransfected with sh-SARA. This suggests that there was a change in the Rab4-positive and Rab11-positive vesicular compartments in EE and RE after SARA suppression.

To study the effect of SARA overexpression, neurons in culture were transfected with a plasmid driving the expression of a SARA-Flag fusion. Consistently with previous reports [23, 44–46], we found that ectopically expressed SARA distributes in a larger vesicular compartment also positive for Rab5 (Fig 2A–2C). Similar results were obtained with Rab4-GFP (Fig 2F–2H), suggesting that SARA overexpression in neurons results in early endosome expansion, probably due to an excess membrane and homotypic fusion of endosomes.

Endocytic membrane traffic in many cell types has an essential role in directing membrane components to their correct location [6, 47]. Growth, remodeling and maintenance of the axonal and dendritic compartments in neurons rely heavily on membrane trafficking. In this scenario, Rab11 is a key protein involved in the transport of material from peripheral EE to the RE [48–49]. We therefore tested the effect of SARA on recycling endosome distribution and morphology. In cells overexpressing SARA, Rab11-GFP endosomes lost the main juxtanuclear distribution usually observed in control neurons (Fig 2K), and Rab11A-positive recycling endosomes appeared as a scattered fluorescent signal throughout the neuronal soma (Fig 2L and 2M).

**Fig 3:**
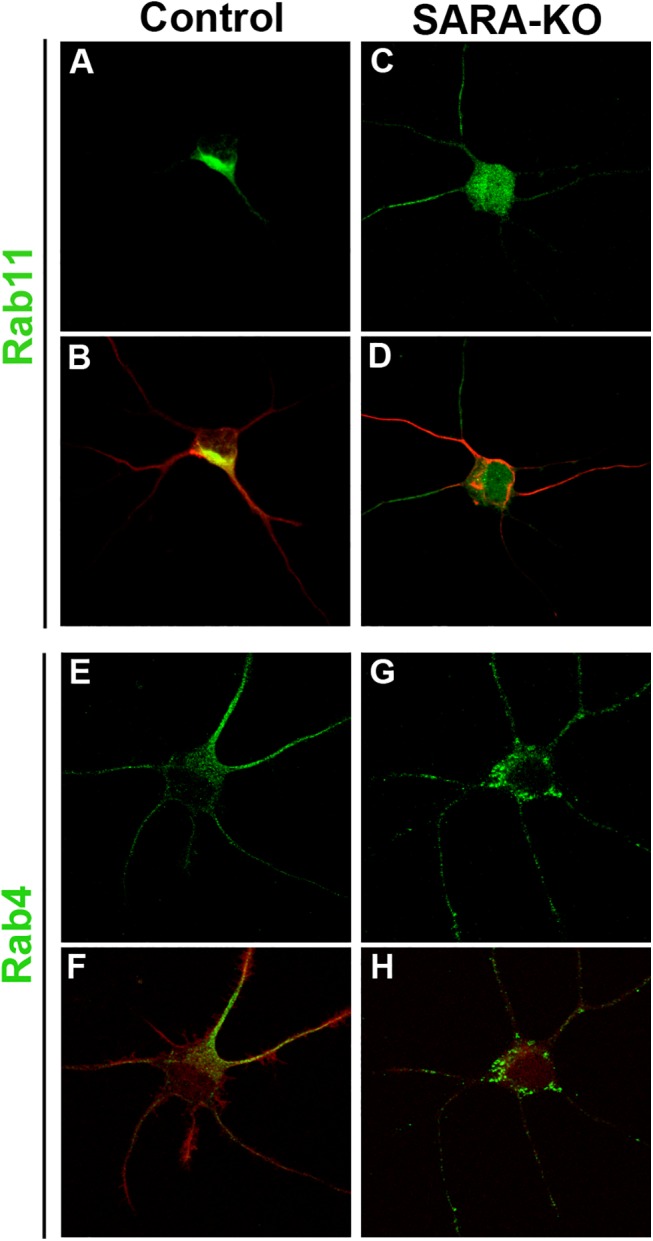
Rab11 and Rab4 positive endosome distribution in SARA-KO neurons. (A-D) Double-stained neurons for Rab11 and Tyr-Tubulin. Tyr-Tubulin labeling is used to show the general morphology of the neuronal cells. (E-H) Double-stained neurons for Rab4 and Rhodamine–Phalloidin. Phalloidin stains F-actin and shows integrity of actin cytoskeleton. Note that Rab11- and Rab4-positive recycling endosomes appear as a scattered fluorescent signal throughout the neuronal soma in SARA-KO neurons (C, G) vs juxtanuclear localization in control neurons (A, E). All cells were imaged by confocal microscopy. Data is typical of over 10 neurons imaged for each condition.

In the next series of experiments, hippocampal neurons in culture were then transfected with a specific shRNA designed to knockdown SARA, (HcRed-tagged sh-SARA; S2 Fig) and the morphology/distribution of EE or RE was analyzed in cells co-expressing Rab5-GFP, or Rab4-GFP or Rab11-GFP constructs. No apparent alterations were detected in EE (Rab5-GFP distribution) in SARA suppressed neurons compared to control neurons (Fig 2A vs. 2D and 2E), but Rab4-GFP endosomes changed their distribution (Fig 2F vs. 2I and 2J; S3 Fig) completely occupying the neuronal perikaryon. In cells where SARA was knocked down, Rab11-positive recycling endosomes also occupied the neuronal perikaryon area, spreading beyond the usual region where they are predominantly founded in control neurons (Fig 2K vs. 2N and 2O). Similar results were found when we analyzed Rab4 and Rab11-endosomes distribution in SARA-depleted neurons from SARA-KO mice (Fig 3).

It is known that Rab5 stimulates the fusion between EEs [50] and that Rab4 activity facilitates the fission of tubules emanating from EEs and preferentially regulates direct recycling from the sorting endosomes [41; 51]. The multiple and dynamic organization of Rab5, Rab4 and Rab11 constitutes a mosaic of membrane domains involved in functional communication between contiguous Rab domains ensuring that the flow of cargo molecules through distinct domains on endosomes is properly regulated [52–53].

Our data suggest a new role for SARA as a regulator of the pathways connecting early, sorting and recycling endosomes into hippocampal neurons. We propose that changes in SARA expression would lead to shifts in those Rab regulated pathways.

### SARA regulates neuronal development

Recycling of the plasma membrane is known to regulate axon extension [54–55]; however, the specific molecules and molecular mechanisms underlying membrane dynamics during neuronal migration and morphogenesis have not been fully characterized. Interestingly, expression of dominant-negative Rab5 mutants enhanced NGF-mediated neurite outgrowth in PC12 cells, whereas a constitutively active Rab5 mutant or Rabex-5 inhibited this process [56].

**Fig 4:**
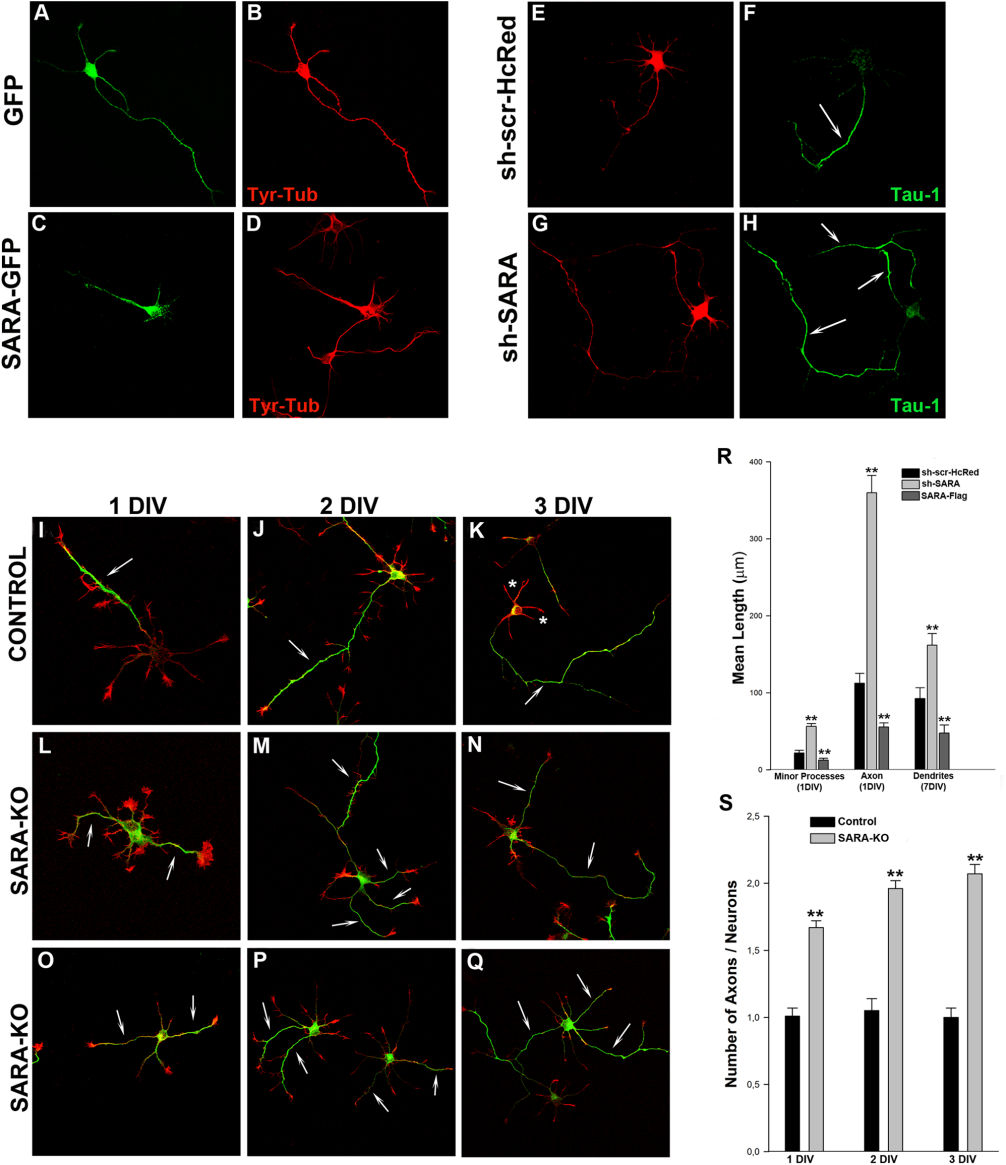
SARA suppression enhances axon elongation and induces formation of supernumerary axons. (Upper panel, left) Morphology of 1 DIV neurons transfected with GFP (A) or SARA-GFP (C), double stained with a mAb against Tyr-Tubulin (B and D). Note that neurons with overexpressed SARA have their neuritic development arrested or delayed. (Upper panel, right) Images showing examples of neurons treated with control sh-scr-HcRed (control; E-F) or sh-SARA (G-H). Cultures were transfected with the corresponding plasmids (1–2 μg DNA each) 24 h after plating and counterstained with a mAb against the axonal marker Tau-1 (green, F-H). Note that SARA suppression increases axonal length and produces the formation of supernumerary axons (arrows in H), contrasting with the single axon in control neurons (arrow in F). Quantification of neurite length in knockdown SARA neurons reveals a significant increase in minor processes, axons and dendrites (**p<0.001; R), respect to the control or SARA overexpressed neurons. For dendrite measurement, neurons were transfected with the same constructs but at 7 DIV and counterstained with a mAb against MAP2, a marker of neuronal dendrites. (Bottom panel) Hippocampal pyramidal neurons from control mice (I-K) or SARA-KO mice (L-Q) fixed at 1, 2 or 3 DIV and stained with a mAb against Tau-1 (green) and Rhodamine-Phalloidin (red). Note that the most of SARA-KO neurons show two or more axons (arrows). Confocal images were from independent experiments, litter mice, and different pregnant females. Bar graph shows average number of Tau-1-positive axons / 50 neurons analyzed (**p<0.001; S).

Gain-of-function studies were subsequently performed to examine the phenotypes of neurons with ectopic expression of SARA. In these experiments, neurons were transfected with SARA-GFP or SARA-Flag and the morphology of the cells was analyzed. The results obtained showed that neurons overexpressing SARA-GFP (Fig 4C and 4D) displayed abnormally short neurites when compared to mock-transfected control cells, expressing either GFP (Fig 4A and 4B) or Flag (not shown). Morphometric analysis revealed a 50% reduction in the length of minor processes, axons and dendrites in neurons over expressing SARA (Fig 4R; Table 1). Thus, SARA overexpression inhibits neurite extension and increases the homotypic fusion of EE.

**Table 1:** Effect of SARA suppression or overexpression on neuronal shape parameters.

	Minor Processes Length 1DIV (μm)	Axonal Length 1DIV (μm)	Dendrites 7DIV (μm)
GFP	21.77 ± 3.51	112.47 ± 12.79	92.60 ± 13.93
sh-SARA	56.45 ± 3.61	359.92 ± 22.39	161.82 ± 15.23
SARA-GFP	12.49 ± 2.51	55.59 ± 5.32	47.66 ± 14.62

Neurons were transfected with 1–2 μg of plasmid for the experiments. Each value represents the mean ± s.e.m. of at least 30 cells for each experimental condition. Statistical analysis was determined as described in Materials and Methods.

To analyze SARA participation in neuronal morphogenesis, we evaluate the consequences of its suppression on neurite outgrowth and axon extension in cultured neurons. To this end, neurons of 1 DIV were transfected with sh-SARA and examined 20–24 h later by costaining with Tau-1 antibody, to identify the axon. The majority (90%) of SARA-silenced neurons (Fig 4G and 4H) reached stage 3 of neuritic development, displaying much longer and branched axon-like neurites (Tau-1 +) than control cells, transfected either with HcRed alone or scrambled sh-RNA (sh-scr-HcRed) (Fig 4E and 4F). Identical results were observed of hippocampal pyramidal neurons cultured from SARA-KO mice, stained with Tau-1 antibody at 1, 2 or 3 DIV. These neurons exhibited two or more Tau-1+ axons (Fig 4I–4Q ans 4S). Quantitative measurements confirmed these observations and revealed that SARA suppression disrupts the polarity of these neurons, resulting in a 2-3-fold increase in the length of minor neurites and number of axons, (Fig 4R; Table 1). Our results suggest that SARA silencing promotes axon and neurite outgrowth and abolishes neuronal polarity.

All together, these results constitute the first indication of SARA being a regulator of neuronal morphology and development.

### SARA suppression perturbs somatodendritic and axonal protein delivery

**Fig 5:**
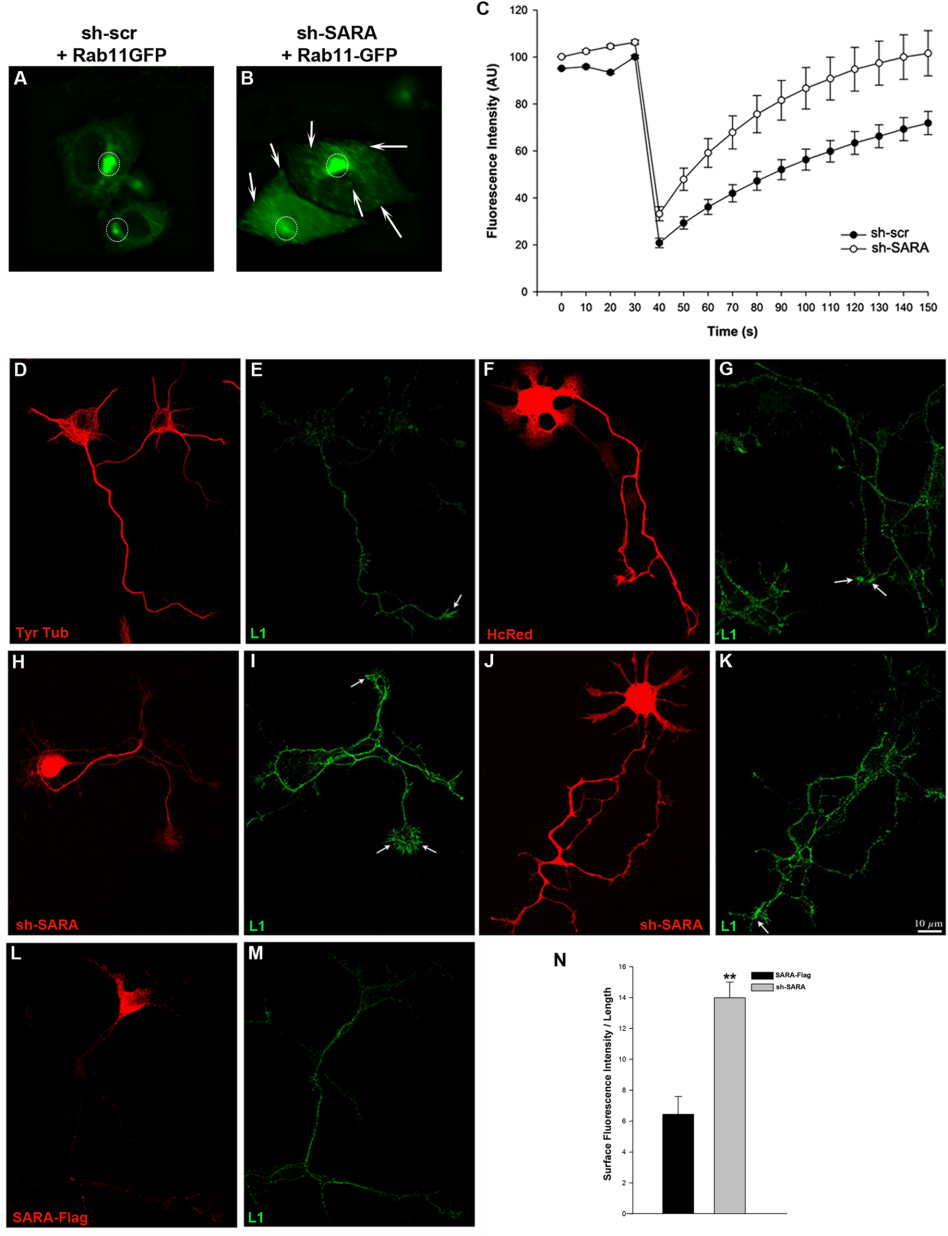
SARA as regulator of membrane delivery. (Upper panel) Images from FRAP experiments in CHO-K1 cells. RE was photobleached in the regions of interest (ROI, circles) in cells expressing Rab11-GFP with sh-scr-HcRed (A) or co-transfected with sh-SARA (B). Arrows indicate Rab11-positive recycling endosomes dispersed over all cells. Recovery of fluorescent molecules from the surrounding area into the photobleached region was monitored over the 150 sec during which the frames were taken. The graph quantifies the recovery into the photobleached area as the fluorescence intensity obtained in three different FRAP experiments (5 control or sh-SARA cells were analyzed in each experiment). SARA suppression significantly increases the movement of Rab11-GFP endosomes into photobleached areas (C). (Bottom panel) Suppression of SARA alters the surface distribution of the axonal membrane protein L1. Confocal images showing the surface distribution of L1: (D-E) control neurons stained for Tyr-Tubulin or transfected with: (F-G) HcRed, (H-K) sh-SARA, (L-M) SARA-Flag. Neurons were exposed to primary antibody against L1 without permeabilization. (H-K) Images show apparent increase of L1 in the axonal membrane compared to the L1 level in control or SARA over-expressed neurons. Quantification of fluorescence intensity (IF) of 3 different regions of the axons (L = 10 μm each region) and the IF/L ratios were averaged to obtain a single value by neuron (n = 12 control and sh-SARA neurons). The graph confirms the observation finding more L1 on the axonal surface of SARA silenced neurons (N) (**p<0.001).

We next assessed whether the enhanced neurite outgrowth observed in SARA-depleted cells could be explained through differential membrane delivery in axonal and somatodendritic compartments. FRAP (fluorescence recovery after photobleaching) experiments were performed to measure the mobility of fluorescent Rab11-positive recycling endosomes and their behavior when SARA was depleted. CHO-K1 cells were transfected using Rab11-GFP + sh-scr-HcRed for control cells (Fig 5A) or Rab11-GFP + sh-SARA (Fig 5B) and analyzed 20h later. The stronger area of Rab11-GFP signal was photobleached by confocal laser microscopy and monitored for fluorescence recovery. These experiments show that, in SARA knockdown cells fluorescence recovery in Rab11-positive recycling endosomes was significantly faster than in control cells (Fig 5C). These data suggest that the imbalance caused by a decrease in the levels of SARA at the EEs may result in a higher number of vesicles directed to the RE or vesicles that move faster to the RE, probably avoiding the regulatory processes mediated by Rabs.

L1 is a cell adhesion molecule highly enriched in the axonal compartment [5], and it has been demonstrated that its endocytosis and trafficking are required for axonal adhesion, growth cone advance and migration [54, 57]. Alterations in RE cause an increase of membrane delivery and an accumulation of L1 at the soma membrane of PC12 cells [58–60]. We therefore analyzed whether the alterations in RE observed after SARA suppression produced changes in L1 endocytosed at the axonal membrane. Neurons were transfected with sh-SARA or HcRed and surface L1 was visualized by incubation of fixed cells with an anti-L1 antibody before detergent extraction. We quantified the amount of L1 protein added at the growth cone periphery, as an indirect measure of the membrane that is aggregated when SARA expression is modified. SARA suppressed neurons showed higher levels of L1 at the axonal and growth cone membranes (Fig 5H–5K) than HcRed controls or SARA-overexpressing neurons (Fig 5D–5G, 5L and 5M, respectively). Quantitative fluorescent measurements confirmed these observations (Fig 5N), suggesting a function of SARA in the regulation of membrane trafficking to the axonal compartment, the main source for its growth and elongation.

**Fig 6:**
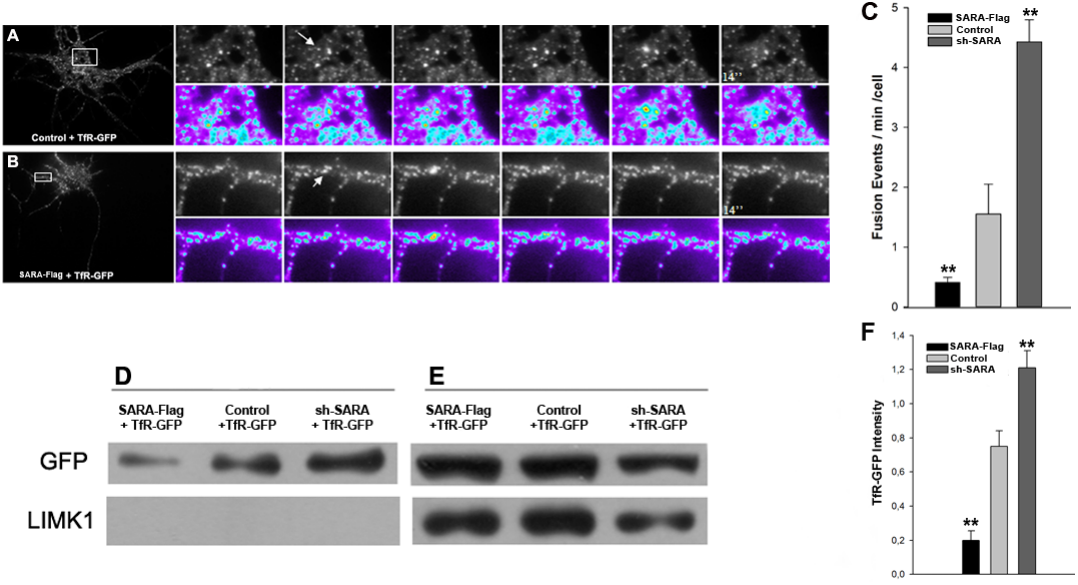
SARA suppression perturbs somatodendritic protein delivery. Time lapse images by TIRFM showing fusion events of vesicles to the plasma membrane in neurons of 7 DIV, transfected with TfR-GFP plus HcRed (A) or SARA (B). Images were taken every 2 seconds for at least 2 minutes. The graphic shows a significant reduction of fusion events at the somatodendritic plasma membrane in cells transfected with SARA plus TfR-GFP and significantly more fusion events in sh-SARA transfected neurons (C; n = 17 neurons; **p<0.001). TheTfR-GFP cell-surface protein increases after SARA silencing. CHO-K1 were transfected with TfR-GFP alone or plus SARA-Flag or sh-SARA. The cultures were incubated with the membrane-impermeable sulfo-NHS-biotin at 4°C to prevent endocytosis. Then the cells were harvested and biotinylated proteins were collected with avidin-agarose beads (Pierce). Membrane (D) and intracellular (E) proteins were isolated by centrifugation and analyzed by Western Blot with the indicated antibodies. Absence of cytosolic contamination was corroborated with anti-LIMK1 antibody. Graph showing the increase of TfR-GFP fluorescence intensity relative to total protein load (labeled with Tubulin; F) in SARA suppressed neurons respect to control or SARA overexpressed neurons. Data are means of three independent experiments.

To evaluate a role of SARA as a regulator of membrane traffic to the somatodendritic compartment, we assessed the dynamics of transferrin receptor (TfR), a dendritic membrane protein. Selective sorting and delivery are responsible for the dendritic targeting of TfR [61]. We used TIRFM to evaluate how the expression of SARA alters the fusion of TfR-GFP-containing vesicles with the somatodendritic plasma membrane. TfR-GFP fusion events were defined as those vesicles approaching to the plasma membrane (rapid spread), followed by fluorescence scattering and subsquent disappearance of the vesicle. Examples of fusion events are shown in Fig 6A and 6B. Quantification of the total number of fusions every 2 min revealed that SARA overexpression significantly reduced the number of TfR-GFP positive vesicles fusing to the somatodendritic membrane, while in neurons transfected with sh-SARA the number of fusion events were 3–4 fold higher than in control neurons (Fig 6C).

These results were confirmed using a biochemical approach. SARA expression was altered in CHO-K1 cells and cell-surface proteins were labeled with a sulfo-biotin reagent. Cells were cotransfected with SARA-Flag + TfR-GFP or HcRed + TfR-GFP or sh-SARA + TfR-GFP and cell-impermeant sulfo-biotin was added to the culture medium at 4°C, at which temperature the endocytosis was markedly retarded. After biotinylation, cultures were lysed and incubated with avidin beads to obtain two fractions by centrifugation: the pellet with cell-surface biotinylated proteins and the supernatant with intracellular un-biotinylated proteins. Western blot analysis revealed that the total amount of TfR-GFP in membrane was significantly higher in SARA depleted cells than in control or SARA-overexpressing cells (Fig 6D–6F). These experiments show a regulatory effect of SARA on membrane expansion in the somatodendritic and axonal compartments in hippocampal neurons.

## Discussion

In this study, we show a novel and fundamental role for SARA in axon/dendrite development in hippocampal neurons, a phenomenon that probably involves SARA participation in the regulation of endosomal trafficking and membrane expansion. Ectopic expression of SARA leads to inhibition of axonal growth and the formation of enlarged early endosomes. On the other hand, neurons in which SARA was depleted developed multiple and long Tau+ axon-like processes, longer neurites and large growth cones. Furthermore, SARA acute silencing or knockout in culture neurons also disturbed the Rab11-positive recycling endosomes. It is possible that, in neurons where SARA was silenced, there may be higher levels of stable microtubules, thereby producing neurons with multiple axons (polarity alteration) and RE that are more dispersed than in normal conditions. Lin et al. [62] showed that, in cells with a higher level of stable, detyrosinated (Glu) microtubules, the RE were more dispersed, suggesting that stable microtubules play a role in the organization of RE and in facilitating the movement of vesicles from the RE to the cell surface. Consistently, changing the microtubule dynamics is sufficient to alter neuronal polarization. Low doses of the microtubule-stabilizing drug taxol shift polymerizing microtubules from neurite shafts to process tips and lead to the formation of multiple axons [63].

Here we demonstrate that SARA partially colocalizes with EE markers (EEA1, Rab5, and Rab4), which suggests that SARA may play a role linking these domains on EE. Through its interaction with Rab5, SARA could recruit cargo into vesicular clusters of EE and, when interacting with Rab4, it would regulate the entry of cargo to tubular regions of EE to be later directed to the sorting endosomes. Rab proteins associate with different subcompartments generating membrane-specific domains [40]. Rab5 is associated with EEs in which it regulates endocytic vesicle fusion with preexisting EEs and homotypic fusion between early endosomes [64]. During maturation, EEs extend tubules (sorting endosomes Rab4 + [65]) that become the RE. Rab4 distributes into EEs and REs and it has been postulated to control a fast recycling pathway [64; 66–67]. Recently Falk et al. suggested that Rab5 and Rab4 participate in a recycling loop within the growth cone that controls the speed of axon elongation [68]. The present observations are consistent with this proposal and place SARA as a novel regulator of early endosomal trafficking and axon formation in developing neurons.

Our results also suggest a role for SARA in the regulation of Rab11–positive endosomes. Rab11 regulates membrane trafficking between TGN and RE, recycling of TfR [69] and AMPA receptors on the cellular surface in neurons [70]. The existence of a regulated route through various endosomes endows them with the capacity to finely coordinate the distribution of receptors and the extent of signaling [53]. Lazo et al. described how after the addition of BDNF, Rab11-positive structures were less mobile and significantly larger, suggesting that there is an increase of Rab11-positive vesicular compartments in dendrites after dendritic branching stimulation with BDNF [71]. Constitutively active and WT forms of Rab11A significantly increased the axonal outgrowth of cortical neurons [72]. Considering the principal role of active Rab11 in promoting trafficking from recycling endosomes to the plasma membrane, it is conceivable that Rab11-positive vesicular regions or domains observed in SARA-suppressed neurons may function as “Rab11-stations” which stimulate axonal outgrowth by providing axons and dendrites with extra membranes.

Recently, Chang et al. reported that adult SARA–CKO mice (conditional KO) developed normally and described no significant abnormalities compared with WT mice. The histopathological analysis of these animals was not shown in the manuscript [24]. However, reinforcing our findings of neurons transfected with sh-SARA, our results in cultured hippocampal neurons from SARA-KO mice showed supernumerary axons, which imply significant alterations in neuronal polarity. Whether this has an impact on behavior or neurophysiology in the SARA-KO animals remains an open question.

Alterations of SARA expression in neurons not only caused morphological changes but also resulted in functional alterations: SARA silencing resulted in an increased number of TfR containing vesicles that fuse with cellular surface and ectopic SARA expression decreased them. The importance of correct delivery of membrane and its associated proteins for maintenance of the identity of axonal and dendritic compartments in neurons has been recognized [73–74]. Using TfR and L1 proteins, we demonstrate that SARA regulates membrane delivery to somatodendritic and axonal compartments. These effects were also observed in cell lines in which it was proposed that SARA oligomerization could contribute to early endosomal membrane fusion and/or expansion [21].

Together, these findings point up the importance of regulated endosomal trafficking in a membrane expansion process in which SARA would play a role as a new regulator protein. Our data indicated that SARA could work both as a positive regulator of homotypic and heterotypic EE fusion and as a negative regulator of membrane release toward the RE in neurons. We propose that unusually high homotypic endosomes fusion occurs after SARA overexpression, keeping proteins (e.g.TfR) at the EE and thus preventing them being delivered to the RE and plasmatic membrane by the slow recycling pathway or directly to the membrane by the fast recycling pathway. These neurons overexpressing SARA show short neurites and low amounts of TfR and L1 on the surface. On the other hand, in SARA KD /KO neurons, EEs are unable to mature because normal homotypic endosome fusion is necessary for EE maturation, and so there is no regulation/sorting trafficking between EE-RE compartments. It may be that Rab11-Rab4 positive endosomes disperse all over the neuron, which in turn increases TfR (by fast or slow recycling) and L1 (by slow recycling) to the cell surface, generating more axons and larger growth cones.

Finally, the endosomal machinery controls the withdrawal of adhesion molecules in proximal regions and their subsequent insertion at the front of the cell, all of which ensures the traction forces needed to maintain cell morphology and migratory patterns [75]. It has been recently shown that Rab5 and Rab7, two GTPases known to be involved in membrane trafficking and protein recycling control, are important to regulate normal migration [76]. Since SARA activity is downstream of Rab5 direction, it would be interesting to evaluate neuronal migration through in vivo experiments when SARA expression is altered.

Multiple classes of proteins are responsible for ensuring the specificity of the sorting, budding, transport and fusion events during endosomal trafficking in neuronal or non-neuronal cells [77–81]. The endosomal pathway in neurons is not yet completely characterized, but there are many labs actively involved in research and new insights are emerging constantly. The precise roles that various proteins play in neuronal membrane trafficking are being investigated; from now on SARA could be one of these. Our results demonstrate that changes in SARA activity alter neuron development.

## Supporting Information

S1 Fig Colocalization between Rab5-GFP and SARA. Rab5-GFP transfected neurons were stained with SARA antibody. The images (A-C) show partial colocalization. *m1* and *m2* maps generated with Costes colocalization mask (red color in the scale 100% colocalization, blue no colocalization condition; D-E). Similar results for Rab4-GFP transfected neurons (F-G).
(TIF)

S2 Fig SARA expression after sh-SARA treatment. (A) Neurons transfected with sh-SARA (red) for 20hs and followed by immunostaining for SARA (green; B). Arrows show lower expression level of SARA protein in sh-SARA-transfected neuron compared with normal level of SARA (asterisk). Immunoblotting of an equal amount of proteins extracted from neurons treated with SARA-Flag plus control or sh-SARA for 20 hr (C) was revealed using the indicated antibodies.
(TIF)

S3 Fig Rab4-positive endosomes change their distribution after SARA suppression. (A-B) Rab4-GFP endosome localization in control and (C-D) sh-SARA neurons. (B, D) Histograms showing Rab4-positive endosomes flooding the soma in sh-SARA neuron.
(TIF)

## References

[1] Bonifacino JS, Rojas R. Retrograde transport from endosomes to the trans-Golgi network. Nat Rev Mol Cell Biol. 2006; 7: 568–579. 16936697 10.1038/nrm1985

[2] Maxfield FR, McGraw TE. Endocytic recycling. Nat Rev Mol Cell Biol. 2004; 5: 121–132. 15040445 10.1038/nrm1315

[3] Rodriguez-Boulan E, Kreitzer G, Müsch A. Organization of vesicular trafficking in epithelia. Nat Rev Mol Cell Biol. 2005; 6: 233–247. 15738988 10.1038/nrm1593

[4] Horton AC, Ehlers MD. Neuronal polarity and trafficking. Neuron 2003; 40: 277–295. 14556709 10.1016/s0896-6273(03)00629-9

[5] Wisco D, Anderson ED, Chang MC, Norden C, Boiko T, Fölsch H, et al Uncovering multiple axonal targeting pathways in hippocampal neurons. J Cell Biol. 2003; 162: 1317–1328. 14517209 10.1083/jcb.200307069 PMC2173963

[6] Sann S, Wang Z, Brown H, Jin Y. Roles of endosomal trafficking in neurite outgrowth and guidance. Trends Cell Biol. 2009; 19: 317–324. 10.1016/j.tcb.2009.05.001 19540123

[7] Shilo B-Z, Schejter ED. Regulation of developmental intercellular signalling by intracellular trafficking. EMBO J. 2011; 30: 3516–3526. 10.1038/emboj.2011.269 21878993 PMC3181488

[8] Villarroel-Campos D, Gastaldi L, Conde C, Caceres A, Gonzalez-Billault C. Rab-mediated trafficking role in neurite formation. J Neurochem. 2014; 129: 40–48. 10.1111/jnc.12676 24517494

[9] Ramírez OA, Couve A. The endoplasmic reticulum and protein trafficking in dendrites and axons. Trends Cell Biol. 2011; 4: 219–227. 10.1016/j.tcb.2010.12.003 21215635

[10] Horton AC, Ehlers M D. Secretory trafficking in neuronal dendrites. Nat Cell Biol. 2004; 6:585–591. 15232591 10.1038/ncb0704-585

[11] Horton AC, Racz B, Monson EB, Linn AL, Ehlers MD. Polarized secretory trafficking directs cargo for asymmetric dendrite growth and morphogenesis. Neuron 2005; 48: 757–771. 16337914 10.1016/j.neuron.2005.11.005

[12] Wandinger-Ness A, Zerial M. Rab proteins and the compartmentalization of the endosomal system. Cold Spring Harb Perspect Biol. 2014; 6: a022616 10.1101/cshperspect.a022616 25341920 PMC4413231

[13] Perlson E, Maday S, Fu M, Moughamian AJ, Holzbaur ELF. Retrograde axonal transport: pathways to cell death? Trends Neurosci. 2010; 33: 335–344. 10.1016/j.tins.2010.03.006 20434225 PMC2902719

[14] De Vos KJ, Grierson AJ, Ackerley S, Miller CCJ. Role of axonal transport in neurodegenerative diseases. Annu Rev Neurosci. 2008; 31:151–173. 10.1146/annurev.neuro.31.061307.090711 18558852

[15] Corbeel L, Freson K. Rab proteins and Rab-associated proteins: major actors in the mechanism of protein-trafficking disorders. Eur J Pediatr. 2008; 167: 723–729. 10.1007/s00431-008-0740-z 18463892 PMC2413085

[16] Salinas S, Bilsland LG, Schiavo G. Molecular landmarks along the axonal route: axonal transport in health and disease. Curr Opin Cell Biol. 2008; 20: 445–453. 10.1016/j.ceb.2008.04.002 18495455

[17] Tsukazaki T, Chiang TA, Davison AF, Attisano L, Wrana JL. SARA, a FYVE domain protein that recruits Smad2 to the TGFbeta receptor. Cell 1998; 95: 779–791. 9865696 10.1016/s0092-8674(00)81701-8

[18] Burd CG, Emr SD. Phosphatidylinositol(3)-phosphate signaling mediated by specific binding to RING FYVE domains. Mol Cell. 1998; 2: 157–162. 9702203 10.1016/s1097-2765(00)80125-2

[19] Wu G, Chen YG, Ozdamar B, Gyuricza CA, Chong PA, Wrana JL, et al Structural basis of Smad2 recognition by the Smad anchor for receptor activation. Science 2000; 287: 92–97. 10615055 10.1126/science.287.5450.92

[20] Bakkebø M, Huse K, Hilden VI, Forfang L, Myklebust JH, Smeland EB, et al SARA is dispensable for functional TGF-β signaling. FEBS Lett. 2012; 586: 3367–3372. 10.1016/j.febslet.2012.07.027 22819827

[21] Hu Y, Chuang JZ, Xu K, McGraw TG, Sung CH. SARA, a FYVE domain protein, affects Rab5-mediated endocytosis. J Cell Sci. 2002; 115: 4755–4763. 12432064 10.1242/jcs.00177 PMC3899687

[22] Runyan CE, Liu Z, Schnaper HW. Phosphatidylinositol 3-Kinase and Rab5 GTPase inversely regulate the Smad Anchor for Receptor Activation (SARA) Protein independently of Transforming Growth Factor-β1 J Biol Chem. (2012); 287: 35815–35824. 10.1074/jbc.M112.380493 22942286 PMC3476251

[23] Kostaras E, Sflomos G, Pedersen NM, Stenmark H, Fotsis T, Murphy C. SARA and RNF11 interact with each other and ESCRT-0 core proteins and regulate degradative EGFR trafficking. Oncogene. 2013; 32: 5220–5232 10.1038/onc.2012.554 23222715

[24] Chang HM, Lin YY, Tsai PC, Liang CT, Yan YT. The FYVE domain of Smad Anchor for Receptor Activation (SARA) is required to prevent skin carcinogenesis, but not in mouse development. PLoS One 2014; 9: e105299 10.1371/journal.pone.0105299 25170969 PMC4149420

[25] Chuang JZ, Zhao Y, Sung CH. SARA-Regulated Vesicular Targeting Underlies Formation of the Light-Sensing Organelle in Mammalian Rods. Cell 2007; 130: 535–547. 17693260 10.1016/j.cell.2007.06.030 PMC3857750

[26] Chuang J, Yen T, Bollati F, Conde C, Cáceres A, Sung CH. The dynein light chain Tctex-1 has a dynein-independent role in actin remodeling during neurite outgrowth. Dev Cell 2005; 9: 75–86. 15992542 10.1016/j.devcel.2005.04.003 PMC3857739

[27] Kunda P, Paglini G, Kosik K, Quiroga S, Cáceres A. Evidence for the involvement of Tiam-1 in axon formation. J Neurosci. 2001; 21: 2361–2372. 11264310 10.1523/JNEUROSCI.21-07-02361.2001 PMC6762399

[28] Long KE, Asou H, Snider MD, Lemmon V. The role of endocytosis in regulating L1-mediated adhesion. J Biol Chem. 2001; 276: 1285–1290. 11035015 10.1074/jbc.M006658200 PMC2426744

[29] Bisbal M, Conde C, Donoso M, Bollati F, Sesma J, Díaz Añel A, et al Protein kinase D regulates trafficking of dendritic membrane proteins in developing neurons. J Neurosci. 2008; 28: 9297–9308. 10.1523/JNEUROSCI.1879-08.2008 18784310 PMC2648138

[30] Bisbal M, Wojnaki J, Peretti D, Roppolo A, Sesma J, Jausoro I, et al KIF4 mediated anterograde translocation and positioning of ribosomal constituents to developing axons. J Biol Chem. 2009; 284: 9489–9497. 10.1074/jbc.M808586200 19158085 PMC2666601

[31] Paglini G, Kunda P, Quiroga S, Kosik K, Cáceres A. Suppression of radixin and moesin alters growth cone morphology, motility, and process formation in primary cultured neurons. J Cell Biol. 1998; 143: 443–455. 9786954 10.1083/jcb.143.2.443 PMC2132841

[32] Rosso S, Bollati F, Bisbal M, Peretti D, Sumi T, Nakamura T, et al LIMK1 regulates Golgi dynamics, traffic of Golgi-derived vesicles, and process extension in primary cultured neurons. Mol Biol Cell 2004; 15: 3433–3449. 15090620 10.1091/mbc.E03-05-0328 PMC452595

[33] Yudowski GA, Puthenveedu MA, von Zastrow M. Distinct modes of regulated receptor insertion to the somatodendritic plasma membrane. Nat Neurosci. 2006; 9: 622–627. 16604070 10.1038/nn1679

[34] Sinnecker D, Voigt P, Hellwig N, Schaefer M. Reversible photobleaching of enhanced green fluorescent proteins. Biochemistry 2005; 44: 7085–7094. 15865453 10.1021/bi047881x

[35] Trenchi A, Gómez GA, Daniotti JL. Dual acylation is required for trafficking of growth-associated protein-43 (GAP-43) to endosomal recycling compartment via an Arf6-associated endocytic vesicular pathway. Biochem J. 2009; 421: 357–369. 10.1042/BJ20090484 19442238

[36] Sprague BL, McNally JG. FRAP analysis of binding: proper and fitting. Trends Cell Biol. 2005; 15: 84–91 15695095 10.1016/j.tcb.2004.12.001

[37] Runyan CE, Schnaper HW, Poncelet AC. The role of internalization in transforming growth factor beta1-induced Smad2 association with Smad anchor for receptor activation (SARA) and Smad2-dependent signaling in human mesangial cells. J Biol Chem. 2005; 280: 8300–830805. 15613484 10.1074/jbc.M407939200

[38] Conde C, Cáceres A. Microtubule assembly, organization and dynamics in axons and dendrites. Nat Rev Neurosci. 2009; 10:319–332. 10.1038/nrn2631 19377501

[39] Manders EMM, Verbeek FJ, Aten JA. Measurement of co-localization of objects in dual-colour confocal images. J Microsc. 1993; 169: 375–382. 10.1111/j.1365-2818.1993.tb03313.x 33930978

[40] Sönnichsen B, De Renzis S, Nielsen E, Rietdorf J, Zerial M. Distinct membrane domains on endosomes in the recycling pathway visualized by multicolor imaging of Rab4, Rab5, and Rab11. J Cell Biol. 2000; 149: 901–914. 10811830 10.1083/jcb.149.4.901 PMC2174575

[41] Stenmark H. Rab GTPases as coordinators of vesicle traffic. Nat Rev Mol Cell Biol. 2009; 10: 513–525. 10.1038/nrm2728 19603039

[42] van der Sluijs P, Hull M, Webster P, Goud B, Mellman I. The small GTP binding protein rab4 controls an early sorting event on the endocytic pathway. Cell 1992; 70: 729–740. 1516131 10.1016/0092-8674(92)90307-x

[43] Coumailleau F, Fürthauer M, Knoblich JA, González-Gaitán M. Directional Delta and Notch trafficking in Sara endosomes during asymmetric cell division. Nature 2009; 458: 1051–1055. 10.1038/nature07854 19295516

[44] Seet LF, Hong W. Endofin, an endosomal FYVE domain protein. J Biol Chem. 2001; 276: 42445–42454. 11546807 10.1074/jbc.M105917200

[45] Itoh F, Divecha N, Brocks L, Oomen L, Janssen H, Calafat J, et al The FYVE domain in Smad anchor for receptor activation (SARA) is sufficient for localization of SARA in early endosomes and regulates TGF-beta/Smad signalling. Genes Cells 2002; 7: 321–331. 11918675 10.1046/j.1365-2443.2002.00519.x

[46] Panopoulou E, Gillooly DJ, Wrana JL, Zerial M, Stenmark H, Murphy C, et al Early endosomal regulation of Smad-dependent signaling in endothelial cells. J Biol Chem. 2002; 277: 18046–18052. 11877415 10.1074/jbc.M107983200

[47] Pfenninger KH. Plasma membrane expansion: a neuron’s Herculean task. Nat Rev Neurosci. 2009; 10: 251–261. 10.1038/nrn2593 19259102

[48] Ullrich O, Reinsc S, Urbé S, Zerial M, Parton RG. Rab11 regulates recycling through the pericentriolar recycling endosome. J Cell Biol. 1996; 135: 913–924 8922376 10.1083/jcb.135.4.913 PMC2133374

[49] Horgan CP, Hanscom SR, Jolly RS, Futter CE, McCaffrey MW. Rab11-FIP3 links the Rab11 GTPase and cytoplasmic dynein to mediate transport to the endosomal-recycling compartment. J Cell Sci. 2010; 123: 181–191 10.1242/jcs.052670 20026645

[50] Gorvel JP, Chavrier P, Zerial M, Gruenberg J. Rab5 controls early endosome fusion in vitro. Cell 1991; 64: 915–925. 1900457 10.1016/0092-8674(91)90316-q

[51] Chavrier P, van der Sluijs P, Mishal Z, Nagelkerken B, Gorvel JP. Early endosome membrane dynamics characterized by flow cytometry. Cytometry 1997; 29: 41–49. 9298810 10.1002/(sici)1097-0320(19970901)29:1<41::aid-cyto4>3.0.co;2-g

[52] Krauss M, Haucke V. Shaping membranes for endocytosis. Rev Physiol Biochem Pharmacol. 2011; 161: 45–66. 22128406 10.1007/112_2008_2

[53] Huotari J, Helenius A. Endosome maturation. EMBO J 2011; 30: 3481–3500. 10.1038/emboj.2011.286 21878991 PMC3181477

[54] Kamiguchi H, Lemmon V. Recycling of the cell adhesion molecule L1 in axonal growth cones. J Neurosci. 2000; 20: 3676–3686 10804209 10.1523/JNEUROSCI.20-10-03676.2000 PMC1237010

[55] Bonanomi D, Fornasiero EF, Valdez G, Halegoua S, Benfenati F, Menegon A, et al Identification of a developmentally regulated pathway of membrane retrieval in neuronal growth cones. J Cell Sci. 2008; 121:3757–3769. 10.1242/jcs.033803 18940911 PMC2731302

[56] Liu J, Lamb D, Chou MM, Liu JY, Li G. Nerve growth factor-mediated neurite outgrowth via regulation of Rab5. Mol Biol Cell 2007; 18: 1375–1384. 17267689 10.1091/mbc.E06-08-0725 PMC1838971

[57] Kamiguchi H, Yoshihara F. The role of endocytic l1 trafficking in polarized adhesion and migration of nerve growth cones. J Neurosci. 2001; 21: 9194–9203 11717353 10.1523/JNEUROSCI.21-23-09194.2001 PMC6763905

[58] Shirane M, Nakayama KI. Protrudin induces neurite formation by directional membrane trafficking. Science 2006; 314: 818–821. 17082457 10.1126/science.1134027

[59] Yap CC, Nokes RL, Wisco D, Anderson E, Fölsch H, Winckler B. Pathway selection to the axon depends on multiple targeting signals in NgCAM. J Cell Sci. 2008; 121: 1514–1525. 10.1242/jcs.022442 18411247

[60] Yap CC, Wisco D, Kujala P, Lasiecka ZM, Cannon JT, Chang MC, et al The somatodendritic endosomal regulator NEEP21 facilitates axonal targeting of L1/NgCAM. J Cell Biol. 2008; 180: 827–842. 10.1083/jcb.200707143 18299352 PMC2265569

[61] Burack MA, Silverman MA, Banker G. The role of selective transport in neuronal polarity sorting. Neuron 2000; 26:465–472. 10839364 10.1016/s0896-6273(00)81178-2

[62] Lin SX, Gundersen GG, Maxfield FR. Export from pericentriolar endocytic recycling compartment to cell surface depends on stable, detyrosinated (Glu) microtubules and kinesin. Mol Biol Cell 2002; 13: 96–109. 11809825 10.1091/mbc.01-05-0224 PMC65075

[63] Witte H, Neukirchen D, Bradke F. Microtubule stabilization specifies initial neuronal polarization. J Cell Biol. 2008; 180: 619–632. 10.1083/jcb.200707042 18268107 PMC2234250

[64] Zerial M, McBride H. Rab proteins as membrane organizers. Nat Rev Mol Cell Biol. 2001; 2: 107–117. 11252952 10.1038/35052055

[65] Grant BD, Donaldson JG. Pathways and mechanisms of endocytic recycling. Nat Rev Mol Cell Biol. 2009; 10: 597–608. 10.1038/nrm2755 19696797 PMC3038567

[66] Maxfield FR, McGraw TE. Endocytic recycling. Nat Rev Mol Cell Biol. 2004; 5: 121–132. 15040445 10.1038/nrm1315

[67] Yudowski GA, Puthenveedu MA, Henry AG, von Zastrow M. Cargo-mediated regulation of a rapid Rab4-dependent recycling pathway. Mol Biol Cell 2009; 20: 2774–2784. 10.1091/mbc.E08-08-0892 19369423 PMC2688556

[68] Falk J, Konopacki FA, Zivraj KH, Holt CE. Rab5 and Rab4 regulate axon elongation in the Xenopus visual system. J Neurosci. 2014; 34: 373–391 10.1523/JNEUROSCI.0876-13.2014 24403139 PMC3870927

[69] Ren M, Xu G, Zeng J, De Lemos-Chiarandini C, Adesnik M, Sabatini DD. Hydrolysis of GTP on rab11 is required for the direct delivery of transferrin from the pericentriolar recycling compartment to the cell surface but not from sorting endosomes. Proc Natl Acad Sci USA. 1998; 95: 6187–6192. 9600939 10.1073/pnas.95.11.6187 PMC27621

[70] Wilcke M, Johannes L, Galli T, Mayau V, Goud B, Salamero J. Rab11 regulates the compartmentalization of early endosomes required for efficient transport from early endosomes to the trans-golgi network. J Cell Biol. 2000; 151: 1207–1220. 11121436 10.1083/jcb.151.6.1207 PMC2190589

[71] Lazo OM, Gonzalez A, Ascaño M, Kuruvilla R, Couve A, Bronfman FC. BDNF Regulates Rab11-Mediated Recycling Endosome Dynamics to Induce Dendritic Branching. J Neurosci. 2013; 33: 6112–6122. 10.1523/JNEUROSCI.4630-12.2013 23554492 PMC3684039

[72] Takano T, Tomomura M, Yoshioka N, Tsutsumi K, Terasawa Y, Saito T, et al LMTK1/AATYK1 Is a Novel Regulator of Axonal Outgrowth That Acts via Rab11 in a Cdk5-Dependent Manner. J Neurosci. 2012; 32: 6587–6599. 10.1523/JNEUROSCI.5317-11.2012 22573681 PMC6621118

[73] Park M, Penick EC, Edwards JG, Kauer JA, Ehlers MD. Recycling endosomes supply AMPA receptors for LTP. Science 2004; 305: 1972–1975. 15448273 10.1126/science.1102026

[74] Park M, Salgado JM, Ostroff L, Helton TD, Robinson CG, Harris KM, et al Plasticity-induced growth of dendritic spines by exocytic trafficking from recycling endosomes. Neuron 2006; 52: 817–830. 17145503 10.1016/j.neuron.2006.09.040 PMC1899130

[75] Shieh JC, Schaar BT, Srinivasan K, Brodsky FM, McConnell SK. Endocytosis regulate cell soma translocation and the distribution of adhesion proteins in migrating neurons. PLoS ONE 2011; 6: e17802 10.1371/journal.pone.0017802 21445347 PMC3062553

[76] Kawauchi T, Sekine K, Shikanai M, Chihama K, Tomita K, Kubo K, et al Rab GTPases-dependent endocytic pathways regulate neuronal migration and maturation through N-cadherin trafficking. Neuron 2010; 67: 588–602. 10.1016/j.neuron.2010.07.007 20797536

[77] Kennedy MJ, Ehlers MD. Organelles and trafficking machinery for postsynaptic plasticity. Annu Rev Neurosci. 2006; 29: 325–362. 16776589 10.1146/annurev.neuro.29.051605.112808 PMC1876664

[78] Dittman J, Ryan TA. Molecular circuitry of endocytosis at nerve terminals. Annu Rev Cell Dev Biol. 2009; 25: 133–160. 10.1146/annurev.cellbio.042308.113302 19575674

[79] Winckler B, Mellman I. Trafficking guidance receptors. Cold Spring Harb Perspect Biol. 2010; 2: a001826 10.1101/cshperspect.a001826 20504966 PMC2890194

[80] Itofusa R, Kamiguchi H. Polarizing membrane dynamics and adhesion for growth cone navigation. Mol Cell Neurosci. 2011; 48:332–338. 10.1016/j.mcn.2011.03.007 21459144

[81] Lasiecka ZM, Winckler B. Mechanisms of polarized membrane trafficking in neurons—focusing in on endosomes. Mol Cell Neurosci. 2011; 48: 278–287. 10.1016/j.mcn.2011.06.013 21762782 PMC3205304

