# Supplementary figures and images for: Involvement of SARA in Axon and Dendrite Growth
Source: PLoS One. 2015 Sep 25;10(9):e0138792. doi: 10.1371/journal.pone.0138792 (PMC4583221; doi:10.1371/journal.pone.0138792)

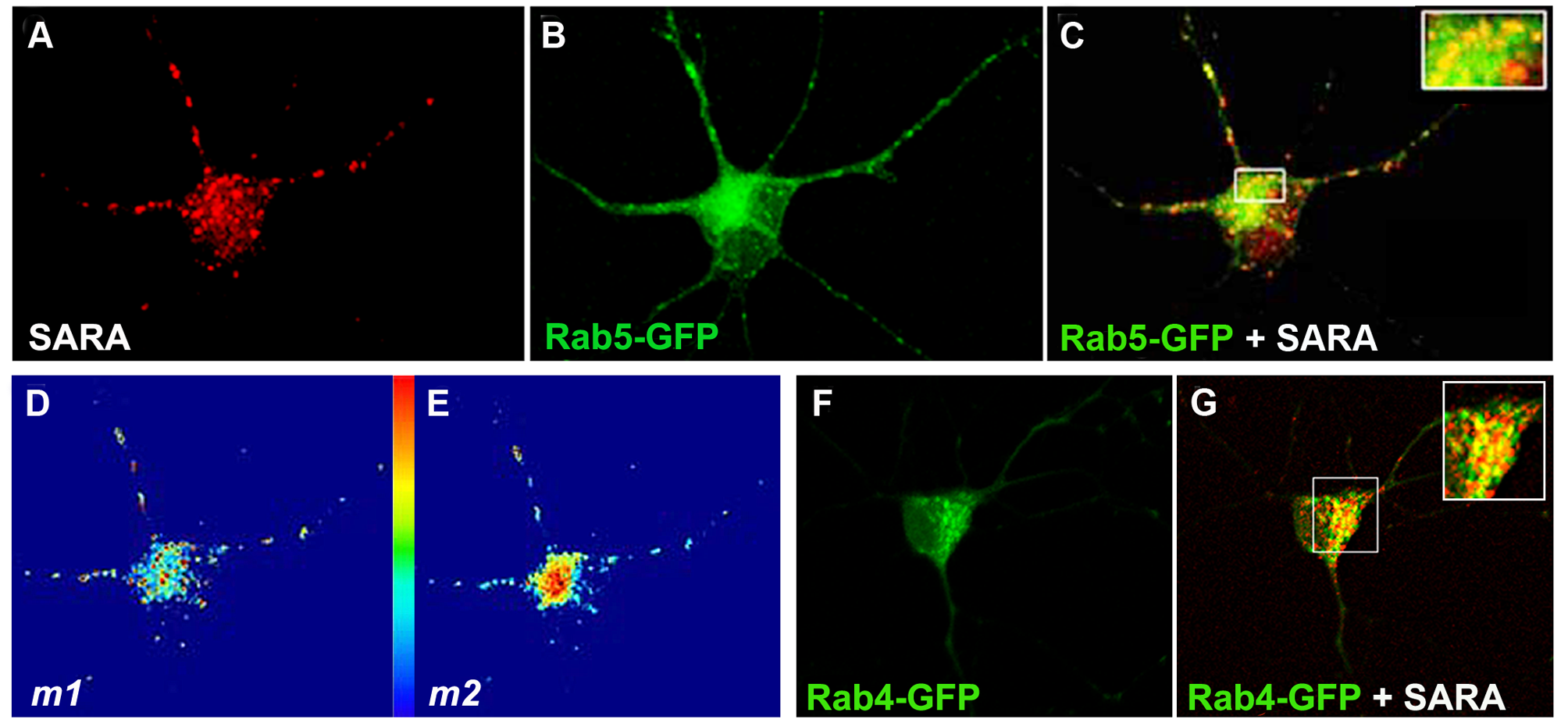

Supplement: S1 Fig — Rab5-GFP transfected neurons were stained with SARA antibody. The images (A-C) show partial colocalization. m1 and m2 maps generated with Costes colocalization mask (red color in the scale 100% colocalization, blue no colocalization condition; D-E). Similar results for Rab4-GFP transfected neurons (F-G). (TIF) [file pone.0138792.s001.tif]

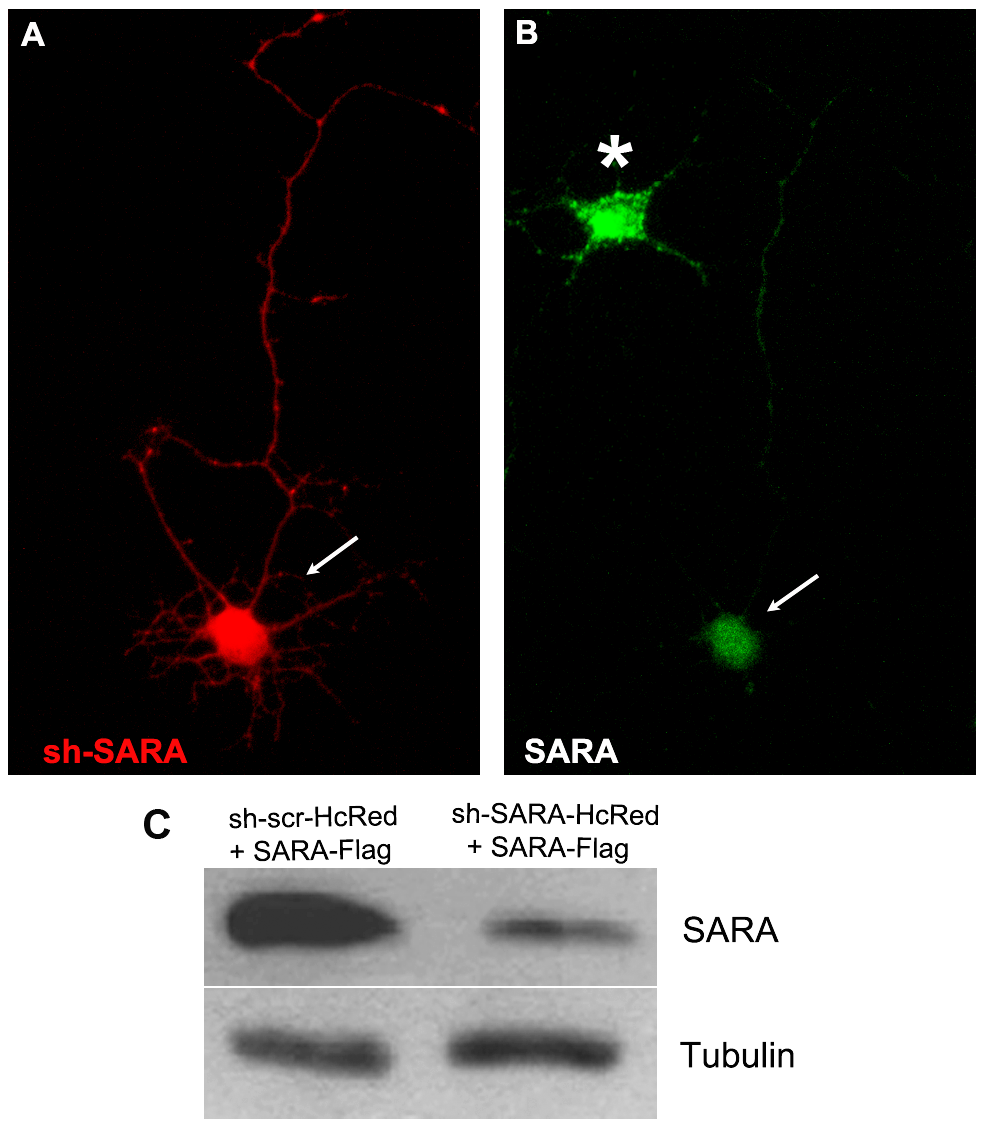

Supplement: S2 Fig — (A) Neurons transfected with sh-SARA (red) for 20hs and followed by immunostaining for SARA (green; B). Arrows show lower expression level of SARA protein in sh-SARA-transfected neuron compared with normal level of SARA (asterisk). Immunoblotting of an equal amount of proteins extracted from neurons treated with SARA-Flag plus control or sh-SARA for 20 hr (C) was revealed using the indicated antibodies. (TIF) [file pone.0138792.s002.tif]

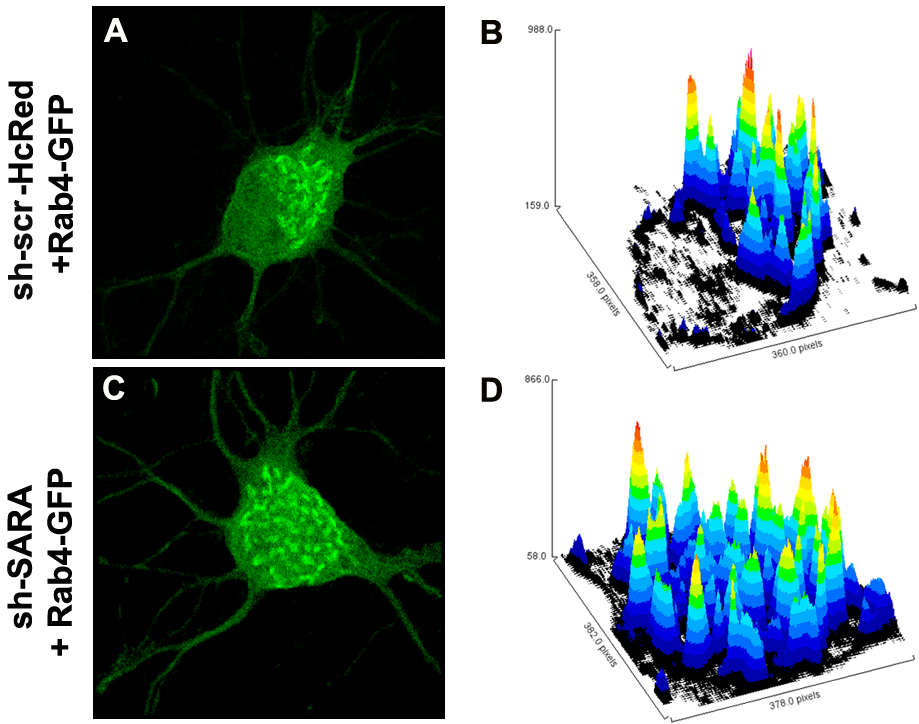

Supplement: S3 Fig — (A-B) Rab4-GFP endosome localization in control and (C-D) sh-SARA neurons. (B, D) Histograms showing Rab4-positive endosomes flooding the soma in sh-SARA neuron. (TIF) [file pone.0138792.s003.tif]
